# High-accuracy pseudospectral scheme for fractional diffusion-wave problems based on cardinal functions

**DOI:** 10.1371/journal.pone.0353233

**Published:** 2026-07-30

**Authors:** Behzad Nemati Saray, Davron Aslonqulovich Juraev, Mohamed Abdalla, Rakib Efendiev, Yahya Almalki, Samad Ahdiaghdam

**Affiliations:** 1 Department of Mathematics, Institute for Advanced Studies in Basic Sciences (IASBS), Zanjan, Iran; 2 Scientific Research Center, Baku Engineering University, Baku, Azerbaijan; 3 Laboratory of Dynamical Systems and Their Applications, Institute of Mathematics Uzbekistan Academy of Sciences, Tashkent, Uzbekistan; 4 Scientific Research Center, Institute of Military Aviation of the University of Military Security and Defense of the Republic of Uzbekistan, Karshi, Uzbekistan; 5 Mathematics Department, Faculty of Science, Qena University, Qena, Egypt; 6 Department of Mathematics and Computer Science, Baku Engineering University, Baku, Azerbaijan; 7 Department of Mathematics, College of Science, King Khalid University, Abha, Saudi Arabia; 8 Department of Mathematics, Marand Branch, Islamic Azad University, Marand, Iran; Government College University Faisalabad, PAKISTAN

## Abstract

Fractional diffusion-wave equations (FDWEs) are essential for modeling complex physical phenomena with memory and hereditary properties, such as anomalous diffusion and viscoelasticity, which classical integer-order models fail to capture accurately. In this paper, we introduce an efficient and high-accuracy pseudospectral scheme utilizing Legendre cardinal functions (LCFs) as basis functions to solve both second- and fourth-order FDWEs. By reformulating the governing equations into equivalent integral forms and developing direct matrix representations for the Caputo fractional derivative and fractional integral operators, we systematically transform the original problem into a solvable system of algebraic equations. Detailed convergence analysis and numerical experiments confirm that this method consistently achieves spectral convergence. A key novelty of this technique, and what significantly advances it beyond previous efforts in the literature, is its exploitation of the cardinal properties of LCFs to avoid numerical integral evaluations when computing basis coefficients entirely. Consequently, this approach dramatically reduces computational overhead while delivering superior accuracy and efficiency compared to existing finite difference and collocation methods.

## 1 Introduction

Fractional differential equations (FDEs) have become a focal point of contemporary mathematical modeling due to their ability to capture complex physical phenomena exhibiting memory and hereditary properties. Among these, fractional diffusion-wave equations (FDWEs) are of particular importance, as they generalize the classical diffusion and wave equations by incorporating derivatives of non-integer order. These equations are applicable in various scientific and engineering fields, including viscoelasticity, anomalous diffusion, signal processing, and subsurface transport. Unlike integer-order models, which often oversimplify dynamic systems, fractional models provide a more accurate description of processes where the rate of change depends not only on the current state but also on the entire history of the system. Recent advances in the theory of fractional calculus have facilitated the development of new analytical and numerical methods to solve such problems. However, numerical treatment of fractional partial differential equations (PDEs) remains challenging due to their non-local nature and the computational complexity involved in approximating fractional operators. Most existing numerical schemes, such as finite difference or finite element methods, require fine discretization to achieve acceptable accuracy, often leading to large-scale algebraic systems and high computational costs. Furthermore, their convergence properties may deteriorate when applied to problems with high-order spatial derivatives or strong temporal singularities. Given the limitations of traditional approaches, there is a growing demand for more efficient and accurate numerical methods for solving FDWEs. Spectral and pseudospectral methods, known for their exponential convergence properties in solving smooth problems, offer a promising alternative. Among these, the use of Legendre cardinal functions (LCFs) stands out due to their interpolation properties, which eliminate the need for integral evaluations in the computation of basis coefficients. This work addresses a timely and important need in applied mathematics: the development of a computationally efficient and highly accurate scheme for solving second- and fourth-order FDWEs. Moreover, fractional models are increasingly used in real-world applications such as seismic wave simulation, heat conduction in heterogeneous materials, and bioengineering systems. Therefore, having a robust and generalizable numerical method that provides reliable solutions to FDWEs contributes directly to advances in modeling such phenomena, reinforcing the practical value and cross-disciplinary impact of this research. The scientific novelty of this work lies in the development of a high-accuracy pseudospectral method based on Legendre cardinal functions for solving both second- and fourth-order fractional diffusion-wave equations. Unlike traditional methods, the proposed approach introduces matrix representations of Caputo fractional derivatives and integrals, eliminating the need for numerical integration of basis coefficients. This significantly improves computational efficiency. Additionally, the method is applicable to a wide range of problems, provides spectral convergence, and offers greater accuracy compared to existing numerical schemes. Its flexibility and rigorous theoretical foundation mark a novel contribution to the numerical analysis of fractional partial differential equations.

In recent years, fractional differential equations (FDEs) have emerged as powerful tools to model complex physical phenomena that cannot be adequately described by classical integer-order derivatives [1–5]. Due to their growing importance, FDEs have attracted significant research interest, leading to advancements in both theoretical development and numerical solutions. Various computational methods have been proposed to solve FDEs efficiently, including the implicit integration factor method [6], the Adomian decomposition method [7], and adaptive grid techniques to enhance numerical accuracy [8]. Wavelet-based methods [9–11] and B-spline collocation approaches [12,13] have also gained popularity for their flexibility in approximating solutions. Additionally, second-order accurate difference schemes [14], finite difference method [15], multistep schemes [16], the classical finite element method [17], compact difference scheme [18], collocation method [8, 19–22], Adaptive-grid technique [23], method of lines and the Petrov-Galerkin finite element-meshfree formulation [24] have been developed to address FDEs. Also, many other applications of fractional order problems can be found in [25–30].

The time-fractional diffusion-wave equation (FDWE) is a mathematical model describing a wide range of physical phenomena. It generalizes the classical diffusion-wave equation by replacing the second-order time derivative with a fractional derivative of order η, where (1<η≤2). This equation models mechanical, acoustic, and electromagnetic behaviors [31]. Furthermore, fourth-order spatial derivatives appear in wave propagation within beams and surface groove development, making the fourth-order FDWE a subject of extensive research [32]. This article builds upon previous foundational works related to ill-posed problems, spectral analysis, and boundary value problems. Specifically, it extends the regularization techniques and analytical approaches to Cauchy problems for elliptic systems developed in [33–36], where various methods for solving first-order elliptic systems in bounded and unbounded domains were proposed. The spectral framework applied in the present study is also influenced by the spectral analysis of non-self-adjoint differential operator pencils and branching structures explored in [37,38], providing a theoretical basis for operator behavior in complex domains. In addition, the treatment of wave equations and quantum systems in bounded domains, as discussed in [39], underpins the use of advanced differential formulations. Finally, the Fredholm properties of periodic and general boundary value problems examined in [40,41] inform the mathematical treatment of boundary conditions and ensure the robustness of the proposed numerical schemes. His study also draws upon recent advancements in numerical methods for differential equations. In particular, the modified Gauss quadrature techniques for solving initial-value problems for ODEs proposed by Ibrahimov and Imanova [42], as well as their approaches to increasing the accuracy of numerical solutions in applied problems [43], have been influential in shaping the discretization strategies and precision analysis adopted in this work. We consider a mathematical model defined over a rectangular domain in space and time, where the unknown function describes the dynamic behavior of a physical system. The evolution of this function is governed by a fractional time derivative of Caputo type, with the order of differentiation lying between one and two, capturing both memory effects and intermediate behavior between diffusion and wave propagation. The equation includes a second-order spatial derivative, representing the distribution of the quantity across space. Additionally, the system is influenced by a source function that varies in both spatial and temporal dimensions. To ensure a well-posed problem, we impose initial conditions, specifying the state and the initial rate of change of the function at the beginning of the time interval. We also enforce boundary conditions at the spatial endpoints, controlling the values of the solution along the edges of the spatial domain. This formulation is particularly relevant in applications involving anomalous transport, viscoelastic materials, or signal propagation in complex media.

This work focuses on solving the second-order FDWE [44–48]


$$\begin{array}{c} \overset{C}{\underset{0}{\;}}{𝒟}_{t}^{\eta }(u)(s,t)={𝒟}_{s}^{2}(u)(s,t)+q(s,t),(s,t)\in [0,1]\times [0,1], \end{array}$$ (1)

with initial-boundary conditions


$$\begin{array}{c} \left\{ \begin{array}{c} u(s,0)=\phi_{0}(s),\;{𝒟}_{t}(u)(s,0)=\phi_{1}(s),\;s\in [0,1],\\ u(0,t)=f_{0}(t),\;u(1,t)=f_{1}(t),\;t\in [0,1],\; \end{array}\right. \end{array}$$

and the fourth-order FDWE,


$$\begin{array}{c} \overset{C}{\underset{0}{\;}}{𝒟}_{t}^{\eta }(u)(s,t)+{𝒟}_{s}^{4}(u)(s,t)=q(s,t),(s,t)\in [0,1]\times [0,1], \end{array}$$ (2)

with conditions


$$\begin{array}{c} \left\{ \begin{array}{c} u(s,0)=\phi_{0}(s),\;{𝒟}_{t}(u)(s,0)=\phi_{1}(s),\;s\in [0,1],\;\\ u(0,t)=f_{0}(t),\;u(1,t)=f_{1}(t),\;t\in [0,1],\;\\ {𝒟}_{s}^{2}(u)(0,t)=f_{1}(t),\;{𝒟}_{s}^{2}(u)(0,t)=f_{1}(t),\;t\in [0,1] \end{array}\right. \end{array}$$

where $\overset{C}{\underset{0}{\;}}{𝒟}_{t}^{\eta }$ indicates the Caputo derivative, ${𝒟}_{s}=\frac{\partial }{\partial s}$, and η∈(1,2]. Here we assume that *u* and *q* are smooth and continuous functions.

The Caputo fractional derivative in problems (4)-(4) models physical systems where the present state depends on the entire history of evolution, unlike classical integer-order models that capture only instantaneous change. This behavior appears in wave propagation through viscoelastic media, anomalous diffusion in porous or heterogeneous materials, and seismic and biological signal propagation. Therefore, fractional diffusion-wave equations allow capturing intermediate behavior between pure diffusion and classical wave dynamics, which cannot be accurately described by standard models.

Various numerical approaches have been applied to solve such equations, including the spectral tau method [44], the implicit difference scheme [45], high-order compact finite difference methods [46,47], the finite difference scheme [48], a compact difference scheme [49, 50], collocation method [51], the separating variables [52] and the Sumudu transform method [53].

The pseudospectral method, a prominent member of the spectral method family, offers high accuracy in solving differential equations due to its interpolation-based approach. Unlike finite difference methods, which rely on local discretization, the pseudospectral scheme approximates solutions using collocation points. This method leverages orthogonal polynomials to achieve rapid convergence, particularly for problems with smooth solutions, and ensures high accuracy by minimizing residuals at collocation points. These advantages make it well-suited for complex problems, including high-dimensional and nonlinear equations [54].

Numerical approximation of FDWEs is challenging due to the non-local nature of fractional derivatives, often resulting in dense coefficient matrices and high computational complexity. To address these limitations, we introduce a pseudospectral scheme based on Legendre cardinal functions. Compared to finite-difference and finite-element methods, spectral techniques provide higher accuracy with fewer degrees of freedom for smooth solutions. Unlike existing spectral approaches that rely on operational matrices requiring numerical integration, the present method exploits cardinal functions to eliminate integral evaluations and produce direct matrix representations of fractional operators. This leads to a highly accurate and computationally efficient scheme that significantly improves performance over existing methods.

The remainder of this paper is organized as follows: Section 2 introduces Legendre cardinal functions (LCFs) and their key properties, along with matrix representations of fractional integral and Caputo fractional derivative operators. Section 3 details the numerical scheme for solving FDWEs using the pseudospectral approach, including convergence analysis. Section 4 presents numerical experiments and results. Finally, Section 5 provides concluding remarks and summarizes the study’s key findings.

## 2 Legendre Cardinal functions

The eigenfunctions of the Sturm-Liouville problem:


$$\begin{array}{c} {𝒟}_{s}\left(\left(1-s^{2}\right){𝒟}_{s}\left(u(s)\right)\right)+\lambda u(s)=0,\;{𝒟}_{s}=\frac{d}{ds}. \end{array}$$

Are known as Legendre polynomials. These polynomials are associated with eigenvalues $\lambda_{n}=n(n+1)$, where *n* is a non-negative integer. A closed-form expression for these polynomials is given by:


$$\begin{array}{c} P_{n}(s)=\frac{1}{2^{n}}\sum_{j=0}^{n}(\begin{array}{c} n\\ j \end{array}{)}^{2}(s+1{)}^{j}(s-1{)}^{n-j}. \end{array}$$ (3)

This formula, derived from Rodrigues’ representation, ensures $P_{n}(1)=1$. Additionally, Legendre polynomials satisfy the three-term recurrence relation:


$$\begin{array}{c} P_{0}(s)=1,\;P_{1}(s)=s, \end{array}$$

$$\begin{array}{c} (n+1)P_{n+1}(s)=(2n+1)sP_{n}(s)-nP_{n-1}(s),\;n\geq 1. \end{array}$$

These polynomials are orthogonal with respect to the *L*^2^ inner product over [−1,1].


$$\begin{array}{c} {\left(P_{n}(x),P_{n^{{\;}^{\prime }}}(s)\right)}_{2}=\int_{-1}^{1}P_{n}(x)P_{n^{{\;}^{\prime }}}(s)ds=\frac{2}{2n+1}\delta_{n,n^{{\;}^{\prime }}}, \end{array}$$

where $\delta_{n,n^{{\;}^{\prime }}}$ is the Kronecker delta function.

To extend Legendre polynomials to an arbitrary domain [a,b], we apply the transformation:


$$\begin{array}{c} P_{n}^{*}(s)=P_{n}\left(\frac{2(s-a)}{b-a}-1\right),\;n=0,1,\ldots ,N. \end{array}$$ (4)

Since Legendre polynomial roots lack closed-form expressions, numerical methods such as eigenvalue techniques or iterative approaches are used. These roots are real, distinct, and lie within [−1,1]. The corresponding roots for shifted Legendre polynomials are computed as:


$$\begin{array}{c} s_{n}^{*}=a+\frac{\left(s_{n}+1\right)(b-a)}{2},\;n=0,1,\ldots ,N. \end{array}$$ (5)

Consider the set of nodes $\{s_{n}\overset{N}{\underset{n=0}{\}}}$ as the roots of $P_{N+1}$. The corresponding Legendre cardinal functions are defined by:


$$\begin{array}{c} c_{n}(s)=\frac{P_{N+1}^{*}(s)}{D_{s}\left(P_{N+1}^{*}\left(s_{n}^{*}\right)\right)\left(s-s_{n}^{*}\right)},\;n=0,1,\ldots ,N,\;s\in [a,b]. \end{array}$$ (6)

Another approach to defining these functions involves selecting a grid based on the extrema of the polynomial $P_{N}^{*}(s)$, supplemented with the interval endpoints. This grid, known as the Lobatto grid, is described as:


$$\begin{array}{c} \{s_{n}^{*}\overset{N}{\underset{n=0}{\}}}=\left\{\left\{\overset{*}{\underset{n}{\hat{s}}}\overset{N-2}{\underset{n=0}{\}}}\cup \left\{a,b\right\}\right|\;D_{s}{\left(P_{N}^{*}(s)\right)|}_{\{\overset{*}{\underset{n}{\hat{s}}}\overset{N}{\underset{n=0}{\}}}}=0\right\}. \end{array}$$

For nodes selected from the Lobatto grid, the Legendre cardinal functions take the form:


$$\begin{array}{c} c_{n}(s)=\frac{\left(1-s^{2}\right)D_{s}\left(P_{N}^{*}(s)\right)}{D_{s}\left(\left(1-{s_{n}^{*}}^{2}\right)D_{s}\left(P_{N}^{*}\left(s_{n}\right)\right)\right)\left(s-s_{n}^{*}\right)},\;n=0,1,\ldots ,N,\;s\in [a,b]. \end{array}$$ (7)

**Fig 1:**
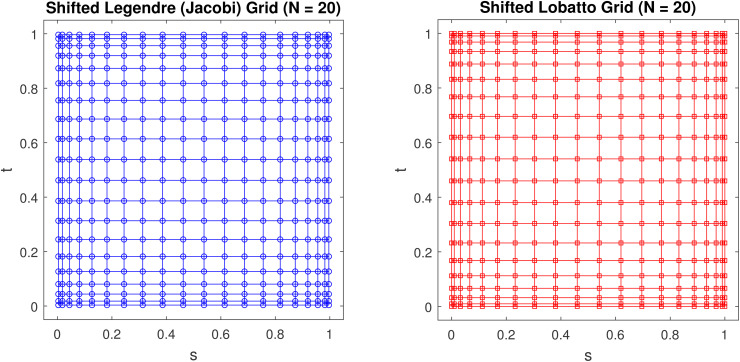
Geometric representation of the shifted Legendre and Lobatto grids on the domain [0,1] ⁎ [0,1].

To visualize the spatial distribution of these nodes, Fig 1 provides a geometric representation of the shifted Legendre and Lobatto grids on the domain [0,1]×[0,1]. Unlike uniform grids, these grids naturally cluster near the boundaries. This clustering is a fundamental geometric property that bounds the interpolation Lebesgue constant, ensuring the spectral accuracy and stability of the proposed scheme.

The fundamental property of these functions is that they are cardinal, i.e.,


$$\begin{array}{c} c_{n}\left(s_{\overset{―}{n}}^{*}\right)=\delta_{n,\overset{―}{n}},\;n,\overset{―}{n}=0,1,\ldots ,N. \end{array}$$ (8)

This property ensures that an *N* degree polynomial precisely interpolates data at *N* + 1 given points. It follows from [8] that any function sampled at *N* + 1 locations can be represented as:


$$\begin{array}{c} u(s)\approx {𝒬}_{N}\left(u(s)\right)=\sum_{n=0}^{N}u\left(s_{n}^{*}\right)c_{n}(s), \end{array}$$ (9)

where ${𝒬}_{N}:L^{2}[a,b]\to \Pi_{N}[a,b]$ acts as a projection mapping, where it projects functions from the *L*^2^ space onto the space of polynomials of degree at most *N*, denoted by $\Pi_{N}[a,b]$.

Given Ω=[a,b]×[0,τ], assume that $\Pi_{N}^{2}(\Omega )$ denotes the space of polynomials of degree at most *N* in both *s* and *t*. Using Legendre cardinal functions, a two-dimensional function $u(s,t)\in L^{2}(\Omega )$ can be approximated as:


$$\begin{array}{c} u(s,t)\approx {𝒬}_{N}\left(u(s,t)\right)=\sum_{n=0}^{N}\sum_{n^{{\;}^{\prime }}=0}^{N}u_{n,n^{{\;}^{\prime }}}c_{n}(s)c_{n^{{\;}^{\prime }}}(t)\in \Pi_{N}^{2}(\Omega ), \end{array}$$ (10)

where the coefficients are computed by $u_{n,n^{{\;}^{\prime }}}=u\left(s_{n}^{*},t_{n^{{\;}^{\prime }}}^{*}\right)$.

Now, consider the weight function:

$w^{\alpha ,\beta }(s)=(1-s{)}^{\alpha }(1+s{)}^{\beta }$, for β>−1.

By defining the transformed weight functions:


$$\begin{array}{c} {\tilde{w}}^{\alpha ,\beta }(s)=w^{\alpha ,\beta }\left(\frac{2(s-a)}{b-a}-1\right),\;{\tilde{w}}^{\alpha ,\beta }(t)=w^{\alpha ,\beta }\left(\frac{2t}{\tau }-1\right), \end{array}$$

we introduce the two-dimensional weight function:


$$\begin{array}{c} {\tilde{𝐰}}^{\alpha ,\beta }(s,t)={\tilde{w}}^{\alpha_{1},\beta_{1}}(s)\times {\tilde{w}}^{\alpha_{2},\beta_{2}}(t). \end{array}$$

The function space ${𝐁}_{\alpha ,\beta }^{m}(\Omega )$ is introduced as in [55], equipped with the norm and semi-norm:


$$\begin{array}{c} |u\overset{2}{\underset{{𝐁}_{\alpha ,\beta }^{m}(\Omega )}{|}}=\sum_{j=1}^{2}\sum_{𝐦\in {ℱ}_{j}}\overset{2}{\underset{{\tilde{𝐖}}^{\alpha +m_{j}{𝐞}_{j},\;\beta +m_{j}{𝐞}_{j}\;}}{\left\|{𝒟}_{𝐬}^{𝐦}(u)\right\|}}, \end{array}$$

$$\begin{array}{c} \overset{2}{\underset{{𝐁}_{\alpha ,\beta }^{m}(\Omega )}{\left\|u\right\|}}=\overset{2}{\underset{{\tilde{𝐖}}^{\alpha ,\beta }}{\left\|u\right\|}}+|u\overset{2}{\underset{{𝐁}_{\alpha ,\beta }^{m}(\Omega )}{|}}, \end{array}$$ (11)

in which 𝐬=(s,t), $\alpha =\left(\alpha_{1},\alpha_{2}\right)$, $\beta =\left(\beta_{1},\beta_{2}\right)$, and ${𝐞}_{j}$ represents the unit vectors in each coordinate direction. Furthermore, we have


$$\begin{array}{c} {ℱ}_{j}=\left\{𝐦\in {\mathbb{N}}_{0}^{2}|2\leq m_{j}\leq m,\;m_{i}\in \{0,1\},\;i\text{≠}j,\;\sum_{j^{{\;}^{\prime }}=1}^{2}m_{j^{{\;}^{\prime }}}=m\right\},\;1\leq j\leq 2. \end{array}$$

Throughout, *C* denotes a generic positive constant, which may vary between equations.

**Theorem 1.** *[56, Theorem 8.6]. If* $u\in {𝐁}_{0,0}^{m}(\Omega )$ *and* 2≤m≤N+1*, then the error in the polynomial approximation satisfies:*


$$\begin{array}{c} ∥{𝒬}_{N}(u)-u{∥}_{L^{2}(\Omega )}\leq C\sqrt{\frac{(N+1-m)!}{N!}}(N+m{)}^{-(m+1)/2}{\left(\frac{k_{\max}}{2}\right)}^{2m}|u{|}_{{𝐁}_{0,0}^{m}(\Omega )}, \end{array}$$ (12)

where $k_{\max}=\max\{\tau ,b-a\}$.

### 2.1 Matrix representation of the derivative operator

This section introduces a framework for introducing a matrix that is used to represent the derivative operative based on LCFs. As established in the literature (see, e.g.,), the leading coefficient of $P_{N+1}(s)$ is given by


$$\begin{array}{c} \kappa_{N}=\frac{\Gamma (2N+1)}{(b-a{)}^{N}\Gamma (N+1{)}^{2}}. \end{array}$$ (13)

Differentiating this polynomial yields


$$\begin{array}{c} {𝒟}_{s}^{k}\left(P_{N+1}^{*}\right)(s)=\rho_{N+1,k}P_{N+1-k}^{*}(s), \end{array}$$ (14)

where the coefficient $\rho_{N+1,k}$ is given by


$$\begin{array}{c} \rho_{N+1,k}=\frac{\Gamma (N+k)}{2^{k-1}(b-a)\Gamma (N)}. \end{array}$$ (15)

Utilizing equations (13) and (15), one can write an alternative representation of the LCFs (6) and (7) as


$$\begin{array}{c} c_{n}(s)=\sigma \prod_{j=0,j\text{≠}n}^{N}\left(s-s_{j}^{*}\right),\;n=0,1,\ldots ,N,\;s\in [a,b], \end{array}$$ (16)

where $\sigma =\frac{\kappa_{N}}{{𝒟}_{s}{\left(P_{N+1}^{*}(s)\right)}_{s=s_{n}^{*}}},$ and $\sigma =\frac{-4\rho_{N+1,1}\Gamma (2N-1)}{(N-2)(b-a{)}^{N}\Gamma (N+1){𝒟}_{s}{\left(\left(1-s^{2}\right){𝒟}_{s}\left(P_{N}^{*}(s)\right)\right)}_{s=s_{n}^{*}}},$ for [6] and [7], respectively.

Differentiating both sides of [16] results in


$$\begin{array}{c} {𝒟}_{s}\left(c_{n}\right)(s)=\sigma \sum_{\begin{array}{c} l=0\\ l\text{≠}n \end{array}}^{N}\prod_{\begin{array}{c} j=0\\ j\text{≠}n,l \end{array}}^{N}\left(s-s_{j}^{*}\right)=\sum_{\begin{array}{c} l=0\\ l\text{≠}n \end{array}}^{N}\frac{c_{n}(s)}{\left(s-s_{l}^{*}\right)},\;n=0,1,\ldots ,N. \end{array}$$ (17)

If ${𝒟}_{s}\left(c_{n}(s)\right)$ is approximated in terms of LCFs $c_{n}(s)$, it follows that


$$\begin{array}{c} {𝒟}_{s}\left(c_{n}(s)\right)\approx \sum_{n^{{\;}^{\prime \prime }}=0}^{N}{𝒟}_{s}{\left(c_{n}(s)\right)}_{s=s_{n^{{\;}^{\prime \prime }}}^{*}}c_{n^{{\;}^{\prime \prime }}}(s),\;n=0,1,\ldots ,N. \end{array}$$ (18)

Combining equations (17) and (18) leads to


$$\begin{array}{c} {𝒟}_{s}{\;c_{n}(s)|}_{s_{n^{{\;}^{\prime \prime }}}^{*}}=\{\begin{array}{cc} \sum_{\begin{array}{c} l=0\\ l\text{≠}n \end{array}}^{N}{\left(s_{n^{{\;}^{\prime \prime }}}^{*}-s_{l}^{*}\right)}^{-1}, & n=n^{\prime \prime },\\ \sigma \prod_{\begin{array}{c} j=0\\ j\text{≠}n,n^{{\;}^{\prime \prime }} \end{array}}^{N}\left(s_{n^{{\;}^{\prime \prime }}}^{*}-s_{j}^{*}\right), & n\text{≠}n^{\prime \prime }. \end{array}\} \end{array}$$ (19)

Now, consider the vector function $\Psi_{N}(s)$, whose elements are defined by


$$\begin{array}{c} {\left[\Psi_{N}\right]}_{n+1}(s)=c_{n}(s),\;n=0,1,\ldots ,N. \end{array}$$ (20)

Using this vector notation, the derivative operator can be expressed in matrix form as


$$\begin{array}{c} {𝒟}_{s}\left(\Psi_{N}(s)\right)\approx D\Psi_{N}(s), \end{array}$$ (21)

where the entries of matrix $D\in {\mathbb{R}}^{\left(N+1)\times (N+1\right)}$ are specified by equation (19).

### 2.2 Matrix representation of the fractional integral operator

To express the fractional integral operator (FIO) in matrix form, it is first necessary to reformulate the LCFs [56]. Specifically, we have


$$\begin{array}{c} \prod_{\begin{array}{c} j=0\\ j\text{≠}n \end{array}}^{N}\left(s-s_{j}^{*}\right)=\sum_{i=0}^{N}\varrho_{n,i}s^{N-i}, \end{array}$$ (22)

where the coefficients are defined as


$$\begin{array}{c} \begin{array}{cc} \varrho_{n,0}=1,\; & {\;\varrho }_{n,i}=\frac{1}{i}\sum_{k=0}^{i}v_{n,k}\varrho_{n,i-k},\;v_{n,k}=\sum_{\begin{array}{c} l=0\\ l\text{≠}n \end{array}}^{N}{\left(s_{l}^{*}\right)}^{k},\;n,i=0,1,\ldots ,N. \end{array} \end{array}$$

As a result, the LCFs can also be expressed in an alternative form


$$\begin{array}{c} c_{n}(s)=\sigma \sum_{i=0}^{N}\varrho_{n,i}s^{N-i},\;n=0,1,\ldots ,N,\;s\in [a,b]. \end{array}$$ (23)

Now, two cases can be considered as follows:

- **Case 1:** If *a* = 0, utilizing the definition of the FIO [57], it follows that

$$\begin{array}{c} {𝔗}_{a}^{\eta }\left(s^{\vartheta }\right)=\frac{\Gamma (\vartheta +1)}{\Gamma (\vartheta +\eta +1)}s^{\eta +\vartheta },\;\eta \in R_{+},\;s\in [a,b]. \end{array}$$ (24)

- Using [23] and the reformulated expression in [8], it results that

$$\begin{array}{c} \begin{array}{c} {𝔗}_{a}^{\eta }\left(s^{\vartheta }\right)=\sigma {𝔗}_{a}^{\eta }\left(\sum_{i=0}^{N}\varrho_{n,i}s^{N-i}\right)=\\ =\sigma \sum_{i=0}^{M}\varrho_{n,i}\frac{\Gamma (N-i+1)}{\Gamma (N-i+\eta +1)}s^{N-1+\eta },\;\eta \;n=0,1,\ldots ,N. \end{array} \end{array}$$ (25)

- **Case 2:** If a≠0, then

$$\begin{array}{c} \begin{array}{c} {𝔗}_{a}^{\eta }(s{)}^{\vartheta }=\frac{1}{\Gamma (\eta )}\int_{a}^{s}{\left(s-\xi \right)}^{\eta -1}\xi^{\vartheta }d\xi =\frac{{\left(s-a\right)}^{\eta }a^{\vartheta }}{\Gamma (\eta )}\int_{0}^{1}{\left(1-t\right)}^{\eta -1}{\left(1-t\left(1-\frac{s}{a}\right)\right)}^{\vartheta }dt=\\ =\frac{(s-a{)}^{\eta }a^{\vartheta }}{\Gamma (\eta )}B(1,\eta {)}_{2}F_{1}\left(-\vartheta ,1;\eta +1;1-\frac{s}{a}\right),\; \end{array} \end{array}$$ (26)

- where *B* represents the Beta function, and _2_ *F*_1_ denotes the hypergeometric function, as defined in [58]. Thus, from [8] and [25], it follows that

$$\begin{array}{c} \begin{array}{cc} {𝔗}_{a}^{\eta }(c_{n}(s)) & =\frac{{\sigma (s-a)}^{\eta }B(1,\eta )}{\Gamma (\eta )}\\ & \times \sum_{l=0,n=0,\ldots ,N}^{N}{\varrho_{n,i}(a)}^{N-i}{\;}_{2}F_{1}\left(-(N-i),1;\eta +1;1-\frac{s}{a}\right). \end{array} \end{array}$$ (27)

Having established these formulations, the matrix representation of the FIO based on LCFs is given by


$$\begin{array}{c} {𝔗}_{a}^{\eta }\left(c_{n}(s)\right)\approx \sum_{n^{{\;}^{\prime }}=0}^{N}I_{a}^{\eta }\left(c_{n}\right)\left(s_{n^{{\;}^{\prime }}}^{*}\right)c_{n^{{\;}^{\prime }}}(x),\;n=0,\ldots ,N, \end{array}$$ (28)

where the coefficients $I_{a}^{\eta }\left(c_{n}\right)\left(s_{n^{\prime }}\right)$ are computed using equations (24) and (26). Consequently, the matrix $I_{\eta }\in R^{\left(N+1)\times (N+1\right)}$ is introduced such that


$$\begin{array}{c} {𝔗}_{a}^{\eta }\left(\Psi_{N}(s)\right)\approx I_{\eta }\Psi_{N}(s), \end{array}$$ (29)

with elements defined by


$$\begin{array}{c} {\left[I_{\eta }\right]}_{n+1,n^{{\;}^{\prime }}+1}={𝔗}_{a}^{\eta }\left(c_{n}\right)\left(s_{n^{{\;}^{\prime }}}^{*}\right),\;n,n^{\prime }=0,\ldots ,N. \end{array}$$ (30)

### 2.3 Matrix representation of the Caputo fractional derivative operator

Consider a fractional order $\eta \in R_{+}$ and define ν=⌈η⌉, where ⌈·⌉ denotes the ceiling function. The Caputo fractional derivative (CFD) operator, denoted as $\overset{C}{\underset{a}{\;}}{𝒟}_{s}^{\eta }$, can be expressed in terms of the fractional integral operator as ${𝔗}_{a}^{\nu -\eta }{𝒟}^{\nu }$ when $\eta \notin N_{0}$. Our objective is to construct a matrix ${𝒟}_{\eta }\in {\mathbb{R}}^{\left(N+1)\times (N+1\right)}$ that satisfies the following relation:


$$\begin{array}{c} \overset{C}{\underset{a}{\;}}{𝒟}_{s}^{\eta }\left(\Psi_{N}(s)\right)\approx {𝒟}_{\eta }\Psi_{N}(s). \end{array}$$ (31)

By substituting ${𝔗}_{a}^{\nu -\eta }{𝒟}^{\nu }$ in place of $\overset{C}{\underset{a}{\;}}{𝒟}_{s}^{\eta }$, we obtain:


$$\begin{array}{c} \overset{C}{\underset{a}{\;}}{𝒟}_{s}^{\eta }\left(\Psi_{N}(s)\right)={𝔗}_{a}^{\nu -\eta }{𝒟}^{\nu }\left(\Psi_{N}(s)\right)\approx {𝒟}^{\nu }{𝔗}_{\nu -\eta }\Psi_{N}(s). \end{array}$$ (32)

Consequently, without requiring additional computations, the matrix representation of the CFD operator is derived as:


$$\begin{array}{c} {𝒟}_{\eta }:={𝒟}^{\nu }𝔗. \end{array}$$ (33)

## 3 Proposed algorithm

To establish our proposed numerical method, we combine Equations (4) and (4), leading to the general formulation:


$$\begin{array}{c} \overset{C}{\underset{0}{\;}}D_{t}^{\eta }(u)(s,t)+(-1{)}^{J/2}D_{s}^{n}(u)(s,t)=q(x,t);\;\;\;J=2,4. \end{array}$$ (34)

Using Lemma 2.22 from [57] and applying the fractional integral to both sides of Equation (34), we derive the corresponding integral equation:


$$\begin{array}{c} u(s,t)-y(s,t)+{𝔗}_{0}^{\eta }\left((-1{)}^{\frac{J}{2}}{𝒟}_{s}^{J}(u)-q\right)(s,t)=0,\;t\in [0,1],\;s\in [0,1], \end{array}$$ (35)

where 1<η≤2, and the function *y*(*s*,*t*) is given by $y(s,t)=tD_{t}(u)(s,0)+u(s,0)$. To construct the proposed numerical scheme, we approxima*t*e the function *u*(*s*,*t*) using Legendre cardinal functions, viz.,


$$\begin{array}{c} u(s,t)\approx {𝒬}_{N}(u)(s,t)=\sum_{n=0}^{N}\sum_{n^{{\;}^{\prime }}=0}^{N}u_{n,n^{{\;}^{\prime }}}C_{n}(s)C_{n^{{\;}^{\prime }}}(t):=\tilde{u}(s,t). \end{array}$$ (36)

Substituting this approximation into Equation (35) yields:


$$\begin{array}{c} u(s,t)-\tilde{y}(s,t)+{𝔗}_{0}^{\eta }\left((-1{)}^{J/2}{𝒟}_{s}^{J}\left(\tilde{u}\right)-\tilde{q}\right)(s,t)=0, \end{array}$$ (37)

where $\tilde{y}(s,t)={𝒬}_{N}(y)(s,t)$ and $\tilde{q}(s,t)={𝒬}_{N}(q)(s,t)$. In matrix form, this equation can be rewritten as:


$$\begin{array}{c} \Psi_{N}^{T}(s)\left(U-Y+{𝒟}^{2}UI_{\eta }-GI_{\eta }\right)\Psi_{N}(t)=0, \end{array}$$ (38)

where $U,Y,G\in {\mathbb{R}}^{\left(N+1)\times (N+1\right)}$, and can be obtained as:


$$\begin{array}{c} \begin{array}{c} \tilde{u}(s,t)=\Psi_{N}^{T}(s)U\Psi_{N}(t),\\ {}[5pt]\tilde{y}(s,t)=\Psi_{N}^{T}(s)Y\Psi_{N}(t),\\ {}[5pt]\tilde{q}(s,t)=\Psi_{N}^{T}(s)G\Psi_{N}(t). \end{array} \end{array}$$

The proposed approach is based on the collocation method, which ensures that the approximate solution satisfies the given equation at selected collocation points. This requires minimizing the residual function $r_{N}(s,t)$ at those points, leading to:


$$\begin{array}{c} r(s,t)=\tilde{u}(s,t)-\tilde{y}(s,t)+{𝔗}_{0}^{\eta }\left((-1{)}^{J/2}D_{s}^{J}\left(\tilde{u}\right)-q\right)(s,t). \end{array}$$ (39)

Selecting nodes $\{s_{n}^{*}\overset{N}{\underset{n=0}{\}}}$ as collocation points and employing the matrix representation of the fractional integral and derivative operators, we derive the following system of linear equations:


$$\begin{array}{c} \tilde{u}\left(s_{n}^{*},s_{n^{{\;}^{\prime }}}^{*}\right)-\tilde{y}\left(s_{n}^{*},s_{n^{{\;}^{\prime }}}^{*}\right)+{𝔗}_{0}^{\eta }\left((-1{)}^{J/2}{𝒟}_{s}^{J}\left(\tilde{u}\right)-q\right)\left(s_{n}^{*},s_{n^{{\;}^{\prime }}}^{*}\right)=0,\;\;1\leq n\leq N. \end{array}$$ (40)

Consequently, this formulation results in the linear system ΓU=G. To apply the boundary conditions, they are enforced by modifying the system as follows:


$$\begin{array}{c} \begin{array}{c} \begin{array}{c} 1,n^{{\;}^{\prime }}={\left[\Psi_{N}^{T}(0)U\right]}_{n^{{\;}^{\prime }}},\;[G{]}_{1,n^{{\;}^{\prime }}}={\left[F_{0}\right]}_{n^{{\;}^{\prime }}},\\ {}[\Gamma U{]}_{2,n^{{\;}^{\prime }}}={\left[\Psi_{N}^{T}(0){{𝒟}^{2}}^{T}U\right]}_{n^{{\;}^{\prime }}},\;[G{]}_{2,n^{{\;}^{\prime }}}={\left[F_{2}\right]}_{n^{{\;}^{\prime }}},\\ {}[\Gamma U{]}_{N-1,n^{{\;}^{\prime }}}={\left[\Psi_{N}^{T}(1){{𝒟}^{2}}^{T}U\right]}_{n^{{\;}^{\prime }}},\;[G{]}_{N-1,n^{{\;}^{\prime }}}={\left[F_{3}\right]}_{n^{{\;}^{\prime }}}, \end{array}\\ {}[\Gamma U{]}_{N,n^{{\;}^{\prime }}}={\left[\Psi_{N}^{T}(1)U\right]}_{nn^{{\;}^{\prime }}},\;[G{]}_{N,n^{{\;}^{\prime }}}={\left[F_{1}\right]}_{n^{{\;}^{\prime }}}, \end{array} \end{array}$$

where $F_{i}\in {\mathbb{R}}^{N^{\prime }+1},\;1=0,\ldots ,3$ are given by:


$$\begin{array}{c} F_{i}\approx {𝒬}_{N}\left(f_{i}\right)(t)=\sum_{n^{{\;}^{\prime }}=1}^{N+1}[F{]}_{n^{{\;}^{\prime }}}C_{n^{{\;}^{\prime }}}(t):=F_{i}^{T}\Psi_{N}(t),\;i=0,\ldots ,3. \end{array}$$

Finally, by vectorizing the matrices Γ, *U*, and *G* into $\overset{―}{\Gamma }$, $\overset{―}{U}$, and $\overset{―}{G}$, respectively, the problem reduces to solving the system:


$$\begin{array}{c} \overset{―}{\Gamma }\overset{―}{U}=\overset{―}{G}. \end{array}$$ (41)

This system is effectively solved with the linsolve function in MATLAB (version 2022) to obtain the unknown coefficients $\left\{u_{n,n^{{\;}^{\prime }}}\right\}$ for $0\leq n,\;n^{\prime }\leq N$. This finishes the development of our suggested numerical approach.



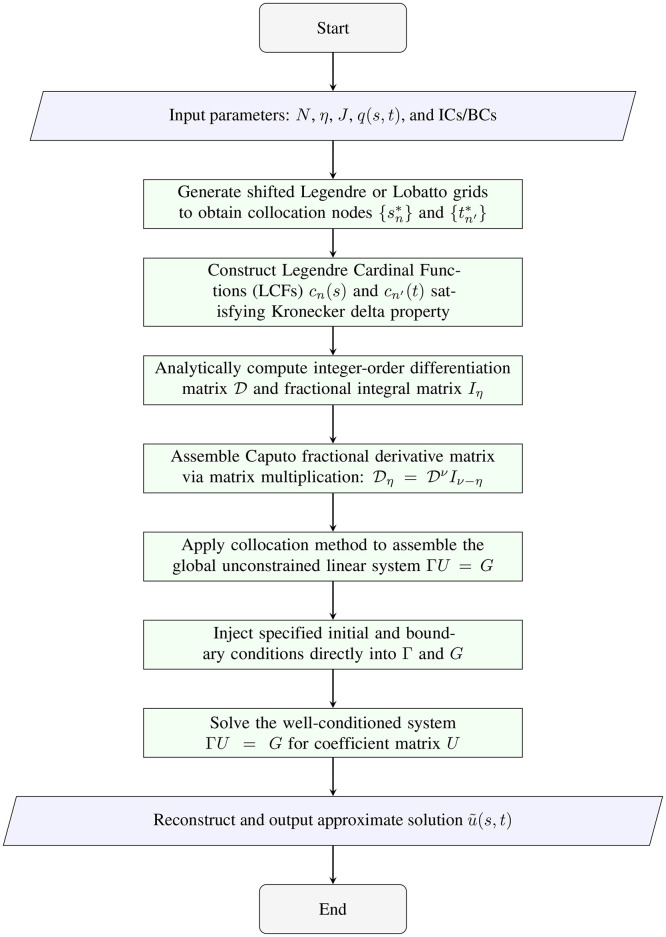



**Remark 1.** *The proposed scheme pre-assembles all operator matrices and stores them for reuse. The differentiation matrix D is formed by evaluating cardinal functions and their derivatives at grid points, which requires* $O\left(N^{2}\right)$ *operations. The fractional integral matrix* $I_{\eta }$*, computed using explicit expressions* [24–26] *also costs* $O\left(N^{2}\right)$*. The Caputo matrix* $D_{\eta }=D^{\nu }I^{\nu -\eta }$ *is obtained via a matrix product with complexity* $O\left(N^{3}\right)$*, performed once. After assembly, the dominant cost is solving the resulting linear system of size (N + 1)*^*2*^*, scaling as* $O\left(N^{6}\right)$ *using direct solvers. Due to spectral convergence, relatively small N values yield high accuracy, making the overall method computationally competitive despite its algebraic scaling.*

### 3.1 Convergence analysis

This section aims to prove that the proposed method is convergence. To this end, the bound introduced in [57] for the FIO will be required. This bound is presented as


$$\begin{array}{c} {\left\|{𝔗}_{0}^{\eta }(u)\right\|}_{L^{p}}\leq \frac{1}{\Gamma (\eta +1)}{\left\|u\right\|}_{L^{p}},\;1\leq p. \end{array}$$ (42)

In more abstract form, the linear system (42) can be written as


$$\begin{array}{c} R(s,t):={𝒬}_{N}\left(r(s,t)\right)=0. \end{array}$$ (43)

Consider $e_{N}=u-\tilde{u}$. Thus, it is not difficult to obtain


$$\begin{array}{c} e_{N}(s,t)+{𝔗}_{0}^{\eta }\left((-1{)}^{J/2}{𝒟}_{s}^{J}\left(e_{N}\right)\right)(s,t)=R(s,t)=\left(T-{𝒬}_{N}\right)r(s,t). \end{array}$$ (44)

Rearrange Equation (44) and taking norm both sides leads to


$$\begin{array}{c} {\left\|e_{N}(s,t)\right\|}_{L^{2}(\Omega )}\leq {\left\|{𝔗}_{0}^{\eta }\left({𝒟}_{s}^{J}\left(e_{N}\right)\right)(s,t)\right\|}_{L^{2}(\Omega )}+{\left\|\left(T-{𝒬}_{N}\right)r(s,t)\right\|}_{L^{2}(\Omega )}. \end{array}$$ (45)

The results of Theorem 1 that


$$\begin{array}{c} \begin{array}{c} ∥e_{N}{∥}_{L^{2}(\Omega )}\leq C\sqrt{\frac{(N+1-m)!}{N!}}(N+m{)}^{-(m+1)/2}{\left(\frac{1}{2}\right)}^{2m}\left(|u{|}_{{𝐁}_{0,0}^{m}(\Omega )}+|r{|}_{{𝐁}_{0,0}^{m}(\Omega )}\right), \end{array} \end{array}$$

Thus, we conclude that


$$\begin{array}{c} ∥e_{N}{∥}_{L^{2}(\Omega )}\to 0, \end{array}$$

as N→∞, provided that $u,q\in {𝐁}_{0,0}^{m}(\Omega )$ and 2≤m≤N+1.

### 3.2 Stability-analysis

This subsection focuses on establishing a stability bound for the proposed pseudospectral method. Recall the integral form of the semi-discrete problem (cf. [48–52]):


$$\begin{array}{c} U(t)-Y(t)+I_{0}^{\eta }\left(AU(t)-G(t)\right)=0,\;t\in [0,T], \end{array}$$ (46)

where $U(t)\in R^{m}$ is the spectral coefficient vector (*m* = (*N* + 1)^2^), *Y*(*t*) and *G*(*t*) are vectors determined by *t*he ini*t*ial data and the source term, and $A\in {\mathbb{R}}^{m\times m}$ is the spatial operator matrix obtained from the differentiation matrices.

**Proposition 1.** Assume that ‖A‖≤M for some constant M≥0, where ∥·∥ denotes the induced 2–norm. Define


$$\begin{array}{c} a(t)=\sup_{0\leq s\leq t}\left(\left\|Y(s)\right\|+\frac{1}{\Gamma (\eta )}\int_{0}^{s}(s-\tau {)}^{\eta -1}\left\|G(\tau )\right\|d\tau \right). \end{array}$$

Then the numerical solution of (46) satisfies


$$\begin{array}{c} \sup_{0\leq s\leq t}\left\|U(s)\right\|\leq a(t)E_{\eta ,1}\left(\frac{Mt^{\eta }}{\Gamma (\eta )}\right),\;t\in [0,T], \end{array}$$

where $E_{\eta ,1}$ denotes the Mittag–Leffler function. In particular, the scheme is stable on [0,T]*.*

*Proof.* Rearranging (46) yields


$$\begin{array}{c} U(t)=Y(t)-I_{0}^{\eta }\left(AU\right)(t)+I_{0}^{\eta }G(t), \end{array}$$

and thus


$$\begin{array}{c} \left\|U(t)\right\|\leq \left\|Y(t)\right\|+\frac{1}{\Gamma (\eta )}\int_{0}^{t}(t-s{)}^{\eta -1}\left\|AU(s)\right\|ds+\frac{1}{\Gamma (\eta )}\int_{0}^{t}(t-s{)}^{\eta -1}\left\|G(s)\right\|ds. \end{array}$$

It follows from


$$\begin{array}{c} \left\|AU(s)\right\|\leq \left\|A\right\|\left\|U(s)\right\|\leq M\left\|U(s)\right\|, \end{array}$$

that


$$\begin{array}{c} \left\|U(t)\right\|\leq \left\|Y(t)\right\|+\frac{M}{\Gamma (\eta )}\int_{0}^{t}(t-s{)}^{\eta -1}\left\|U(s)\right\|ds+\frac{1}{\Gamma (\eta )}\int_{0}^{t}(t-s{)}^{\eta -1}\left\|G(s)\right\|ds. \end{array}$$

Taking into account $u(t)=\sup_{0\leq s\leq t}\left\|U(s)\right\|$, we obtain


$$\begin{array}{c} u(t)\leq a(t)+\frac{M}{\Gamma (\eta )}\int_{0}^{t}(t-s{)}^{\eta -1}u(s)ds. \end{array}$$

Applying the fractional Grönwall inequality (see [59]) yields


$$\begin{array}{c} u(t)\leq a(t)E_{\eta ,1}\left(\frac{Mt^{\eta }}{\Gamma (\eta )}\right). \end{array}$$

This final inequality formally establishes the stability of the proposed scheme. Numerical stability requires that the approximate solutions remain bounded and depend continuously on the problem’s given data. In this formulation, the maximum norm of the numerical solution, *u*(*t*), is strictly controlled by the term *a*(*t*), which contains both the initial conditions *Y*(*t*) and the source term G(τ). The amplification factor is governed by the Mittag-Leffler function $E_{\eta ,1}\left(\frac{Mt^{\eta }}{\Gamma (\eta )}\right)$. Because the Mittag-Leffler function is continuous, finite, and monotonically increasing for any finite time interval t∈[0,T], the numerical solution *U*(*t*) cannot experience unbounded growth provided the initial data and source terms remain bounded. Consequently, the proposed pseudospectral method is unconditionally s*t*able in time on the interval [0, *T*], under the sole spatial condition that the differentiation matrix 𝒜 has a bounded induced 2-norm (||A||≤M).

## 4 Numerical results

Two numerical examples are provided to demonstrate the effectiveness of the presented method. To calculate numerical order of convergence, we fit the exponential model


$$\begin{array}{c} \text{ln}\left(L^{2}-error\right)=a+bN. \end{array}$$

So, the slope *b* tells us the rate of exponential decay. To find *b*, we use least squares


$$\begin{array}{c} b=\frac{\sum (N-N)\left(\text{ln}(e)-\text{ln}(e)\right)}{\sum (N-N{)}^{2}}, \end{array}$$

where $e:=L^{2}-error$ at the given time.

While CPU time offers a practical measure of efficiency, it is inherently hardware-dependent. To provide a robust, machine-independent measure of computational performance, we also evaluated the condition number (κ) of the global collocation matrix $\bar{\Gamma }$. For the test problems, the condition number grows at a manageable polynomial rate with respect to *N*, rather than exponentially. This bounded growth ensures that the linear system in Equation (41) remains well-conditioned and can be solved accurately using standard direct solvers without severe round-off error amplification, further confirming the numerical stability of the proposed LCFs approach.

**Example 4.1**. *Consider the following second-order FDWE:*


$$\begin{array}{c} \left\{ \begin{array}{c} \overset{C}{\underset{0}{\;}}{𝒟}_{t}^{\eta }(u)(s,t)=\frac{1}{2}s^{2}\frac{\partial^{2}u(s,t)}{\partial s^{2}},\;s\in [0,1],\;0<t\leq 1,\;1<\eta \leq 2,\;\;\\ u(s,0)=s,\;{𝒟}_{t}(u)(s,0)=s^{2},\;s\in [0,1],\;\\ u(0,t)=0,\;u(1,t)=\text{sinh}(t),\;t\in [0,1].\; \end{array}\right. \end{array}$$

The exact solution is


$$\begin{array}{c} u(s,t)=s+s^{2}tE_{\eta ,2}\left(t^{\eta }\right), \end{array}$$

in which $E_{\eta ,2}$ specifies the Mittag-Leffler function. For η=2, the exact solution is


$$\begin{array}{c} u(s,t)=s+s^{2}\text{sinh}(t). \end{array}$$

**Fig 2:**
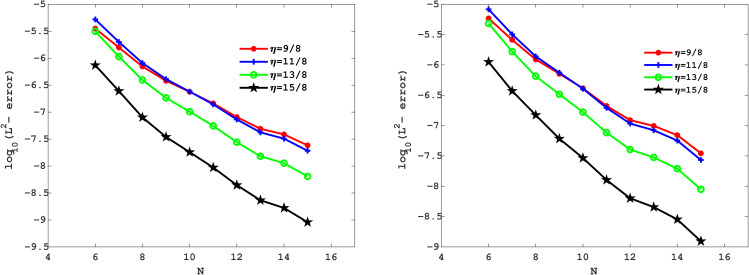
The *L*^2^ errors on a logarithmic scale against the number of basis functions *N* for both the Jacobi (left) and Lobatto (right) grids.

**Fig 3:**
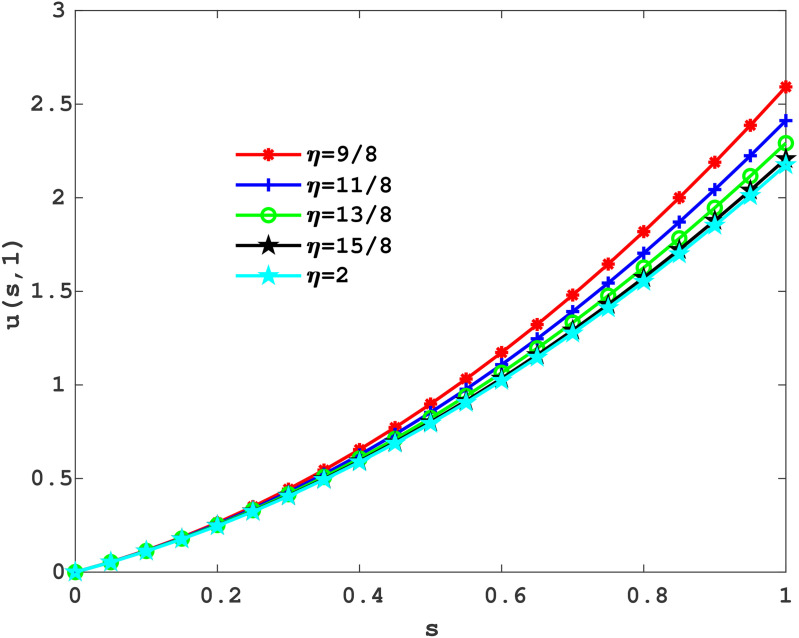
Approximate solution at the final time *t* = 1 for varying choices of the fractional order *η.*

Tables 1 and 2 demonstrate the method’s convergence with the choice of Jacobi and Lobatto grids, and confirm the convergence analysis presented in the previous section. These tables also report the computational time. One can observe that the error decreases as the number of bases *N* increases. Table 3 compares the proposed scheme with the Jacobi collocation method [18]. The results demonstrate that the presented method achieves superior accuracy compared to the Jacobi collocation method. Notably, the proposed method requires lower computational costs due to the cardinality properties of the basis functions. To visually validate the theoretical convergence analysis, Fig 2 plots the *L*^2^ errors on a logarithmic scale against the number of basis functions *N* for both the Jacobi (left) and Lobatto (right) grids. The distinctly linear downward trend observed on these semi-logarithmic plots visually confirms the exponential decay of the error. This steep negative slope demonstrates that spectral convergence is consistently achieved across various fractional orders (η). Furthermore, Fig 3 illustrates the physical behavior of the approximate solution at the final time *t* = 1 for varying choices of the fractional order η. The graph clearly demonstra*t*es a smooth transition in the system’s dynamic response; as the fractional order η→2, the intermediate fractional diffusion-wave profile continuously converges toward the classical, integer-order wave equation solution. This confirms that the proposed pseudospectral scheme accurately captures the physical memory effects parameterized by η. Using the *L*^2^- errors at *t* = 1, we obtain b≈−0.722 for Jacobi grid and b≈−0.651 for Lobatto grid. This indicates exponential decay $O\left(e^{-\text{0.722}N}\right)$ and $O\left(e^{-\text{0.651}N}\right)$ for Jacobi and Lobatto grid, respectively. This confirms spectral convergence, consistent with the theoretical es*t*imate in Theorem 1.

**Table 1:** The *L*^2^ – errors evaluated on the Jacobi grid with different *N* across various times, taking η=1.5.

t\N	6	8	10	12	14
0.1	1.026e−05	8.432e−08	3.744e−07	6.807e−08	2.640e−08
0.2	3.474e−06	7.398e−07	2.039e−07	1.300e−08	2.244e−08
0.3	4.977e−06	7.269e−07	6.150e−08	1.954e−08	1.452e−08
0.4	1.107e−06	9.409e−08	6.806e−08	2.857e−08	1.139e−08
0.5	3.947e−06	5.338e−07	1.071e−07	2.897e−08	9.444e−09
0.6	9.469e−07	6.130e−08	4.832e−08	2.017e−08	7.980e−09
0.7	2.950e−06	4.064e−07	3.316e−08	9.477e−09	6.950e−09
0.8	1.618e−06	2.878e−07	7.245e−08	4.000e−09	6.944e−09
0.9	3.278e−06	1.685e−08	8.075e−08	1.266e−08	4.539e−09
1.0	7.774e−06	1.144e−06	2.440e−07	6.940e−08	2.403e−08
CPU time	0.297	1.641	5.484	19.359	47.531
Condition number	36.9258	155.7334	550.2519	1690	4239.2

**Table 2:** The *L*^2^ – errors evaluated on the Lobatto grid with different *N* across various times, taking η=1.5.

t\N	6	8	10	12	14
0.1	5.613e−06	2.583e−06	6.340e−07	9.575e−08	1.009e−07
0.2	1.482e−05	2.096e−07	5.974e−07	8.573e−08	5.082e−08
0.3	8.662e−06	2.120e−06	3.535e−07	1.443e−09	3.990e−08
0.4	5.439e−06	1.022e−07	1.351e−07	8.191e−08	3.945e−08
0.5	1.073e−05	1.544e−06	3.427e−07	1.003e−07	3.596e−08
0.6	4.530e−06	1.098e−07	9.207e−08	5.692e−08	2.731e−08
0.7	4.823e−06	1.146e−06	1.820e−07	4.686e−10	1.867e−08
0.8	6.507e−06	5.672e−08	2.020e−07	2.760e−08	1.504e−08
0.9	1.498e−06	6.281e−07	1.247e−07	1.743e−08	1.621e−08
1.0	4.838e−08	1.584e−08	3.544e−09	9.009e−10	2.658e−10
CPU time	0.281	1.329	4.812	16.125	43.000
Condition number	53.6772	258.4571	941.9865	2715.6	6607.5

**Table 3:** Maximum absolute errors comparison at *t* = 0.8 between the presented and the Jacobi collocation methods, taking *N* = 8 and various choices of η, for Example 4.1.

	Method	$\eta =\frac{9}{16}$	$\eta =\frac{13}{16}$	$\eta =\frac{15}{16}$
Presented method	Jacobi grid	2.892e−07	2.161e−07	4.708e−08
	Lobatto grid	1.811e−07	1.092e−07	2.283e−08
Other Methods	Jacobi collocation method [?]			
	(α=β=0)	3.55e−7	3.08e−7	1.06e−7
	(α=β=1/2)	3.39e−7	2.96e−7	1.20e−7

**Example 4.2**. *Consider the following fourth-order FDWE,*


$$\begin{array}{c} \overset{C}{\underset{0}{\;}}{𝒟}_{t}^{\eta }(u)(s,t)+{𝒟}_{s}^{4}(u)(s,t)=e^{s}\left(\frac{\Gamma (\eta +4)}{6}t^{3}+t^{\eta +3}+t\right),\;(s,t)\in [0,1]\times [0,1], \end{array}$$

*with conditions*


$$\begin{array}{c} \left\{ \begin{array}{c} u(s,0)=s,\;{𝒟}_{t}(u)(s,0)=0,\;s\in [0,1]\;\\ u(0,t)=t^{\eta +3}+t,\;u(1,t)=e\text{(}t^{\eta +3}+t\text{)},\;\\ {𝒟}_{s}^{2}(u)(0,t)=t^{\eta +3}+t,\;{𝒟}_{s}^{2}(u)(1,t)=e\text{(}t^{\eta +3}+t\text{)},\;t\in [0,1]. \end{array}\right. \end{array}$$

The exact solution is $u(s,t)=e^{s}\text{(}t^{\eta +3}+t\text{)}$ [48].

**Fig 4:**
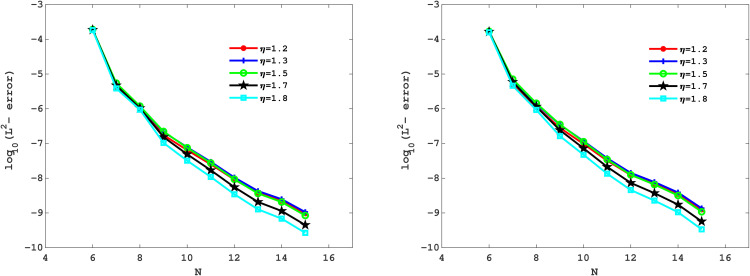
The L^2^ errors obtained from using Jacobi grid% (left) and Lobatto grid (right) for Example 4.2.

Tables 4 and 5 demonstrate the convergence of the proposed method for Jacobi and Lobatto grids, respectively, validating the theoretical convergence analysis. The CPU time is also provided. The results indicate that increasing the number of basis functions *N* reduces the error. Table 6 compares the proposed scheme with the finite difference method [8]. The results show that the proposed approach yields higher accuracy than the finite difference technique. Fig 4 further reinforces the robustness of the proposed scheme for fourth-order spatial derivatives by plotting the *L*^2^ error decay for Example 4.2. Similar to the second-order case, the semi-logarithmic plots for both Jacobi and Lobatto grids exhibit a strict linear decline as *N* increases. This indicates that the exploitation of the cardinal basis functions maintains high-order spectral accuracy and numerical stability even when dealing with the more stringent continuity requirements of fourth-order fractional diffusion-wave equations. Using the *L*^2^- errors at *t* = 1, we obtain b≈−1.155 for Jacobi grid and b≈−3.451 for Lobatto grid. This indicates exponential decay $O\left(e^{-\text{1.155}N}\right)$ and $O\left(e^{-\text{3.451}N}\right)$ for Jacobi and Lobatto grid, respectively. This confirms spectral convergence, consistent with the theoretical estimate in Theorem 1.

**Table 4:** The *L*^2^ – errors evaluated on the Jacobi grid with different *N* across various times, taking η=1.5.

t\N	7	9	11	13	15
0.1	5.891*e* – 06	2.833*e* – 07	5.396*e* – 08	3.384*e* – 10	2.002*e* – 09
0.2	6.475*e* – 06	5.825*e* – 08	3.416*e* – 08	3.994*e* – 09	6.544*e* – 10
0.3	1.115*e* – 06	2.026*e* – 07	2.903*e* – 08	2.938*e* – 09	8.832*e* – 11
0.4	6.145*e* – 06	2.284*e* – 07	1.756*e* – 08	1.460*e* – 09	1.337*e* – 11
0.5	1.541*e* – 06	7.705*e* – 09	4.720*e* – 10	8.176*e* – 11	1.835*e* – 11
0.6	3.395*e* – 06	1.908*e* – 07	1.362*e* – 08	1.097*e* – 09	9.817*e* – 12
0.7	2.491*e* – 06	1.273*e* – 07	1.730*e* – 08	1.636*e* – 09	4.682*e* – 11
0.8	7.255*e* – 06	2.498*e* – 08	1.520*e* – 08	1.591*e* – 09	2.399*e* – 10
0.9	3.126*e* – 06	1.205*e* – 07	1.698*e* – 08	8.932*e* – 11	4.614*e* – 10
1.0	1.712*e* – 05	5.701*e* – 07	5.445*e* – 08	8.220*e* – 09	1.664*e* – 09
CPU time	0.250	0.469	1.297	3.938	8.922
Condition number	47.08	153.88	907.73	1769.20	3798.5

**Table 5:** The *L*^2^ – errors evaluated on the Lobatto grid with different *N* across various times, taking η=1.5.

t\N	7	9	11	13	15
0.1	4.005*e* – 06	7.486*e* – 07	2.346*e* – 08	1.355*e* – 08	2.754*e* – 09
0.2	1.051*e* – 05	5.259*e* – 07	3.388*e* – 08	1.298*e* – 08	3.276*e* – 10
0.3	6.393*e* – 06	2.124*e* – 07	6.270*e* – 08	1.012*e* – 08	7.629*e* – 10
0.4	1.204*e* – 05	5.723*e* – 07	5.142*e* – 08	5.585*e* – 09	4.487*e* – 10
0.5	1.120*e* – 06	1.075*e* – 08	1.097*e* – 09	2.036*e* – 10	4.905*e* – 11
0.6	8.758*e* – 06	4.654*e* – 07	3.964*e* – 08	4.170*e* – 09	3.274*e* – 10
0.7	3.234*e* – 06	1.339*e* – 07	3.682*e* – 08	5.554*e* – 09	3.987*e* – 10
0.8	8.963*e* – 06	2.632*e* – 07	1.472*e* – 08	5.043*e* – 09	1.172*e* – 10
0.9	5.194*e* – 06	2.920*e* – 07	7.092*e* – 09	3.426*e* – 09	6.080*e* – 10
1.0	4.521*e* – 06	1.010*e* – 08	1.222*e* – 11	9.154*e* – 15	4.635*e* – 18
CPU time	0.172	0.391	1.281	3.109	7.890
Condition number	52.61	161.02	867.29	2801.48	7058.5

**Table 6:** Maximum absolute errors comparison at *t* = 1 between the presented and the finite difference methods, taking *N* = 8 and various choices of η, for Example 4.2.

	Method	η=1.3	η=1.5	η=1.8
Presented method	Jacobi grid	3.078*e* – 06	3.516*e* – 06	1.924*e* – 06
	Lobatto grid	2.054*e* – 06	2.038*e* – 06	1.982*e* – 06
Other Methods	Finite difference method [?]			
	τ=1/20	6.9679*e* – 4	2.2181*e* – 3	1.1377*e* – 2
	τ=1/40	2.1513*e* – 4	7.8800*e* – 4	4.993*e* – 3
	τ=1/80	6.6381*e* – 5	2.7933*e* – 4	2.1822*e* – 3

**Example 4.3**. *Consider the following second-order FDWE*


Dtη(u)(s,t)=∂2u(s,t)∂s2+Γ(ρ+1)Γ(ρ+1-η)tρ-η+tρsin(πx), 0C s∈[0,1], 0<t≤1, 1<ρ<η≤2,      u(s,0)=0,   Dt(u)(s,0)=0,   s∈[0,1], u(0,t)=0,   u(1,t)=0,   t∈[0,1].

The exact solution is $u(s,t)=t^{\rho }\text{sin}(\pi x)$. Because ρ−η<0, the Caputo derivative behaves like $t^{\rho -\eta }$ and is singular at *t* = 0. Table 7 is tabulated to show the ability of the method for solving FDWE and illustrate the effect of *t*he weak singularity. As we observe, because the assumed smoothness condition for the problem is violated here, the convergence speed is very slow. However, due to the concentration of Jacobian roots at the origin, the method has managed to solve this type of problem. To achieve better accuracy, we need to increase the number of roots, which will ultimately entail a high computational cost.

**Table 7:** *L*^2^- errors in Jacobi grid, taking η=15/8 and ρ=1.2, for Example 4.3.

	*t* = 0.3	*t* = 0.5	*t* = 0.7	*t* = 0.9
*N* = 16	2.56e−02	2.61e−02	1.56e−02	7.61e−05
*N* = 32	1.65e−02	1.68e−02	1.10e−02	4.18e−05
*N* = 64	6.26e−03	7.87e−03	7.81e−03	2.29e−05

### Comparison with existing methods

Tables 3 and 6 compare the proposed pseudospectral method with representative existing techniques: the Jacobi collocation spectral method [44] and finite-difference schemes for fourth-order FDWEs [48]. For the test problems, the proposed method achieves lower maximum absolute errors at the same number of spatial basis functions *N*. The cardinal Legendre basis’s interpolation properties, which provide spectral-type convergence for smooth solutions, are directly responsible for this behavior. In terms of efficiency, the cardinal property of the basis functions eliminates the need for numerical integration when assembling fractional operator matrices. Consequently, operator matrices are assembled once and reused during computation, reducing assembly overhead compared with methods that compute operational matrices entry-by-entry via numerical quadrature. CPU times reported in Tables 1–6 confirm that the present approach is computationally competitive.

We remark, however, that the principal advantage of our method is most pronounced for problems with smooth spatial dependence, where spectral accuracy reduces required spatial degrees of freedom. For problems exhibiting temporal singularities (e.g., weak initial-time singularity), temporal discretization must be carefully chosen (e.g., graded meshes or nonuniform time-stepping (See, e.g., [60,61]) to preserve convergence.

## 5 Conclusion

This work introduces Legendre cardinal functions and presents a numerical scheme based on the pseudospectral method to solve second and fourth-order fractional diffusion-wave equations. Using Jacobi and Lobatto grids, the Legendre cardinal functions are constructed while satisfying the cardinality condition, a crucial property of such functions.

To reduce computational load, matrix representations of the Caputo fractional derivative (CFD) and fractional integral operators are derived. The proposed scheme transforms the governing equations into corresponding integral equations and solves them using the pseudospectral method. Numerical experiments confirm the theoretical convergence analysis, demonstrating that the method provides highly accurate solutions. Comparisons with existing methods reveal that the proposed approach offers superior accuracy and computational efficiency. Another advantage of this method is its simplicity of implementation. The technique can be extended, with minor modifications, to solve a broad class of linear and nonlinear fractional differential equations, enhancing its applicability in mathematical modeling.

It should be emphasized that the assumptions of smoothness in time near *t* = 0 made in Examples 1–2 may not hold in many realistic fractional‐order applications. When a weak singularity at the initial time is present, specialized time‐ discontinuations (graded, nonuniform, adaptive) are recommended. The present spectral spatial approach is compatible with such time‐stepping strategies and we plan to explore this in future work.

Looking toward future research directions, we plan to extend the proposed Legendre cardinal function framework to address several existing challenges in fractional modeling. First, to mitigate the loss of spectral accuracy caused by weak singularities at the initial time—a common occurrence in realistic fractional-order applications—we intend to integrate our spatial pseudospectral scheme with advanced temporal discretizations, such as graded meshes and adaptive time-stepping algorithms. Second, we aim to generalize this approach to solve nonlinear fractional diffusion-wave equations and extend the mathematical formulation to handle two- and three-dimensional spatial domains. Finally, exploring the application of this method to variable-order fractional models presents a promising avenue, as these models are increasingly required to describe dynamic, time-varying memory effects in highly heterogeneous materials.
